# Environmentally Sustainable Practices in the Operating Room: A French Nationwide Cross-Sectional Survey of Anaesthesiologists and Nurse Anaesthesiologists

**DOI:** 10.5152/TJAR.2022.21410

**Published:** 2022-12-01

**Authors:** Maëlle Tordjman, Cyril Pernod, Lionel Bouvet, Antoine Lamblin

**Affiliations:** 1 Departement of Anaesthesiology and Critical Care, Edouard Herriot Hospital, Lyon University Hospital, Lyon, France; 2 Department of Anaesthesiology, Femme-Mère-Enfant Hospital, Lyon University Hospital, Bron, France

**Keywords:** Anaesthesiology, environment, environmental sustainability, operation room, outpatient anaesthesiology, recycling, waste sorting

## Abstract

**Objective:**

In France, healthcare facilities account for 7% of greenhouse gas emissions and 3.5% of total waste generation. Operating rooms generate 30% of hospital waste and hence should be a primary focus of environmental sustainability initiatives. The aim of this study was to evaluate environmentally sustainable anaesthesiology practices in France in 2020 and understand the barriers to their adoption.

**Methods:**

An anonymous survey of 28 questions was published online. The website did not record participants’ IP addresses. The survey’s link was sent by email to anaesthesiologist and anaesthesia nurse members of the French Society of Anesthesia and Intensive Care Medecine (SFAR), in February and June 2020. The survey was closed in August 2020.

**Results:**

Of the 10 877 recipients, 1092 (10%) responded to the questionnaire. Waste sorting was organized in 69% of respondents’ workplaces (691/1007), and 90% (793/879) of respondents stated that they most often followed the instructions. Sixty-five percent (659/1007) of respondents avoided using the most polluting anaesthetic gases. Thirty-nine percent of respondents (417/1064) had already received environmental sustainability training and 73% (705/972) stated that they wanted more training. The main barriers to the adoption of recycling identified by respondents were staff training (by 70% of respondents, 691/993), budget constraints (66%, 652/993), and a lack of administrative support (60%, 602/993).

**Conclusion:**

French anaesthesiologists and anaesthesia nurses who responded to the survey are environmentally aware and want to improve sustainable practices in the operating room. More widespread adoption could be achieved by offering training to all healthcare professionals and administrative staff and by creating local environmental focus groups to coordinate actions.

**Main Points**

- French anaesthesiologists and anaesthesia nurses are environmentally aware and want to improve sustainable practices in the operating room.
- The barriers to the adoption of recycling would be easy to overcome with better staff training and the creation of local environmental sustainability groups.
- A better integration of environmental issues in hospital policies is also required.

## Introduction

Climate change is a global problem and a considerable danger to populations worldwide.^1,2^ Sustainable development emerged as a concept in 1987 to describe the twin objectives of satisfying present needs while preserving resources for future generations.^3^ A number of international agreements from the Rio Earth Summit in 1992 to the Paris agreement in 2015 have helped to establish national environmental policies and renewable energy targets.

In France, healthcare centres account for 7% of greenhouse gas emissions^4^ and generate 700 000 tons of waste each year, 3.5% of the amount generated nationally.^5^ They also contribute significantly to industrial pollution.^6^

Operating rooms generate 20%-30% of hospital waste and more than 40% of this waste is potentially recyclable.^7,8^ They require a wide range of sterile often single-use medical equipment and consume considerable amounts of water and energy.^9^

Recently, the rising environmental awareness of healthcare professionals has led to the emergence of “green anaesthesia” initiatives aimed at reducing the environmental impact of operating rooms.^10,11^ Potential sustainability improvements in healthcare practices have been widely studied,^12,13^ particularly in the operating room^14,15^ and for waste recycling.^8,16,17^ In anaesthesiology, life cycle analyses have been carried out for anaesthetic gases^18^ and single-use and reusable supplies.^19-21^ Life cycle analyses evaluate the environmental impact of processes or products based on all the resources used from production to disposal.^14^

Surveys have been conducted in several countries to assess sustainability initiatives in operating rooms.^22-24^ In France, the changes in halogenated gas use associated with environmental information campaigns among anaesthesiologists have recently been studied.^25^ However, recycling and environmentally sustainable practices and obstacles to their adoption have never been studied.

The primary objective of this study was to describe existing environmentally sustainable practices in French operating rooms. The secondary objectives were to identify possible barriers to their adoption and highlight potential avenues towards improvements.

## Methods

This study was approved by the ethics committee of the SFAR (IRB approval number: 00010254). The survey was distributed by email in February 2020 to all anaesthesia nurses, anaesthesiologists, and anaesthesia residents in the SFAR research network. A reminder was sent 4 months later, and the survey remained open for 2 months thereafter. The survey was published online by the SFAR at SurveyMonkey.com. It was anonymous and the website did not record participants’ IP addresses.

After conducting a literature review through PubMed on sustainable development practices in the operating room, we also used the guide written by the SFAR green^26^ and a former Canadian survey to develop our questionnaire^22^ (Appendix). It consisted of 28 questions (13 unique choices, 9 multiple choices, 6 Likert scale, and 9 questions with free-response item) organized into 3 parts: (i) demographic characteristics and personal environmentally sustainable practices, (ii) environmental sustainability initiatives in the operating room, and (iii) barriers to their adoption and solutions proposed to overcome them.

The survey was designed by 3 anaesthesiologists trained in ecological issues in the operating room. Once reviewed and validated at local level, it was approved by the environmental sustainability committee of the SFAR.

## Statistical Analysis

The data were retrieved from SurveyMonkey.com and summarized using Microsoft Excel as percentages of respondents. All responses were considered, and whether participants completed the questionnaire in full or not, the number of respondents therefore varied between questions.

## Results

The survey response rate was about 10% (1092 respondents from 10 877 email recipients). The respondents were mostly qualified anaesthesiologists (70%, 765/1092). All categories of the respondents’s characteristics concerning age, geographical location and type of healthcare facility were represented. Participants’ demographic characteristics are summarized in Table 1.

All but 5 respondents (990/995, 99.5%) stated that they tried to limit their environmental impact in their personal life. Ninety-six percent of respondents (952/995) stated that they sorted waste at home. Results for other sustainability practices are reported in Table 2.

Almost all respondents (986/1064, 93%) agreed with the statement that anaesthesiology is a major source of pollution and that practices should account for this. More than 50% of respondents stated that they considered and tried to reduce the environmental impact of anaesthesiology products either all the time (15% of respondents, 146/1007) or often (37%, 372/1007).

Thirty-nine percent of respondents (417/1064) stated that they had already received training on environmental sustainability in anaesthesiology. Apart from informal discussions with colleagues, training was most commonly received during the annual SFAR conference (for 40% of respondents, 192/477). Results for other types of training are listed in Table 3. Only 8% of respondents (87/1064) considered that their knowledge was sufficient to guide their practice.

Regarding environmentally sustainable practices in the operating room, 69% (691/1007) of respondents stated that their place of work had waste sorting facilities. The most commonly recycled materials were paper and cardboard (Table 4).

Almost all respondents (90%, 904/1007) stated that they wanted waste sorting to be improved in their operating room, and 53% (464/879) stated that that they always followed existing waste-sorting instructions.

**Figure 1.**
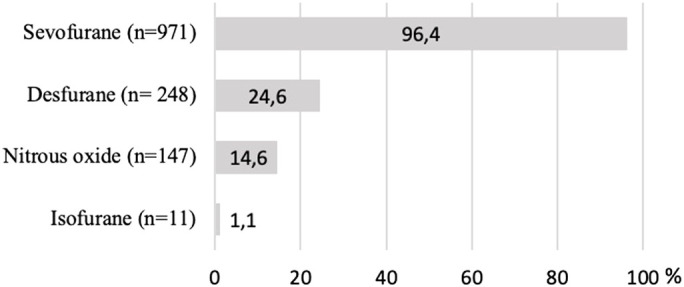
Types of anaesthetic gases used by anaesthesia teams (n = 1007).

Regarding anaesthetic gases, 65% of participants (575/1007) declared that they chose the product with the lowest environmental impact. The most widely used halogenated gas was sevoflurane (Figure 1). Eighty-nine percent of respondents (899/1007) stated that they reduced fresh gas flows in anaesthesia ventilators, 75% used flow rates below 1.5 L min^-1^, and 21% (214/1007) used automated end-tidal control. No respondents declared setting fresh gas flow rates above 3 L min^-1^. The other sustainability practices reported by participants are listed in Table 5.

The main reported obstacles for the adoption of recycling in the operating room were a lack of staff training, a lack of funding, and a lack of support from hospital management (Table 6).

Nearly three quarters of respondents (73%, 705/972) stated that they wanted more information and training on environmental sustainability. The forms of training most desired by respondents (Table 3) were hospital-run programs (by 64% of respondents, 625/972) and expert recommendations (53%, 517/972).

To improve recycling in the operating room, the measure deemed most relevant by respondents was the formation of environmental sustainability groups to initiate actions (highlighted by 84% of respondents, 815/972). The second most selected solution was an increase in training time (41%, 401/972).

## Discussion

To our knowledge, this is the first nationwide study of the environmental sustainability practices of anaesthesiologists, anaesthesia residents, and anaesthesia nurses. The main results are that anaesthesiology teams are aware of the environmental impact of their practices and that they are willing to change them, but there are several barriers to achieving this.

Participants reported much lower rates of waste sorting at work than in their personal life. On top of its environmental benefits, waste sorting is cost-effective for hospitals^8,27^ and improves staff satisfaction.^16^ Healthcare waste management and treatment are costly, but these costs can be reduced by better sorting of contaminated waste, which is 5 times more expensive to treat than ordinary household waste, and by recycling.^9^ Collaborations between healthcare professionals, environmental engineers, sanitation services, and waste management companies are essential to make recycling systems (storage, transport, maintenance) efficient and viable.^5^

Environmental sustainability is also crucial to consider upstream, in procurement, to reduce waste generation. For example, the use of custom packs for a particular treatment reduces the amount of packaging waste overall.^26^ In France, environmental sustainability has been considered a factor since 2010 through the release of purchasing guides^28^ and certifications but is often a secondary concern relative to economic or logistical constraints. Manufacturers should continue to cooperate with providers in optimizing the packaging of healthcare products.

Our results show that anaesthesiologists take account of the environmental impact of anaesthesic gases in their practice, in agreement with another recent study.^25^ These changes have come about thanks to life cycle studies performed in the 2000s and reports by learned societies.^11,26^ Currently, the balance between patient benefit and environmental risk for desflurane and nitrous oxide means that sevoflurane is the anaesthetic gas of choice. The forthcoming availability of capture and recycling systems for halogenated gases may alter practices once more.^14^

The barriers identified here to the adoption of recycling, and by extension sustainability measures in general, are insufficiencies of knowledge, financial means, and administrative support. Staff need to understand the environmental and financial benefits of environmental sustainability to become better engaged. Studies have shown that recycling is improved when staff are provided with information and training.^29^ Guidelines and education resources are available.^11,26^ An environmental sustainability eLearning module has recently been created for French resident anaesthesiologists. University courses are being developed and there are companies specialized in sustainability training (e.g., Committee for Sustainable Development in Health, C2DS). Environmental sustainability courses should be included early in the training of healthcare personnel and should be encouraged by learned societies and hospital management.

Poor environmental awareness at all levels (management, doctors, and paramedics) and the large number of parties involved hinder the implementation of environmentally sustainable practices. This problem could be solved in part by establishing multidisciplinary teams in charge of environmental sustainability in hospitals or hospital networks. These “green teams” would receive specific training on theoretical, practical, and regulatory issues and would raise staff awareness of environmentally sustainable practices and improve communication on these matters between different parties.^30^ Groups such as these have already been created in some hospitals on a voluntary basis, with no specific work time allocation for these activities. Official recognition would facilitate the establishment of these teams and make them more effective.

The absence of administrative support stems from both a lack of staff training and imposed budget constraints. Many healthcare centres are currently facing financial difficulties and are struggling to fulfil their primary role in treating patients. Budget restrictions are guided by short-term objectives centred on healthcare provision. These policies are ill-adapted to considering the long-term health and financial benefits of environmental sustainability initiatives. Furthermore, the regulatory framework in France is complex, poorly understood, and does not encourage sustainability initiatives. A few advances notwithstanding sustainable development policies suffer from the same shortcomings as public healthcare policies, both being subject to chronic underfunding. A recent French parliamentary report on environmental health policy strategies and governance^31^ has highlighted underfunding, the large number of government agencies involved, and a lack of coordinated action as the main barriers to improving the environmental health of the country. These are the same barriers which, at a smaller scale, hinder environmental sustainability initiatives in the operating room.

Hopes for improvement exist with the evolution of the regulatory framework and the increase of incentives at national level. The creation of green teams at local level will allow for better coordination of the various actors. The shortcomings can also be remedied with the help of new government agencies and associations whose mission is to support establishments and professionals in implementing sustainable development.^32,33^

The main limitations of this study are the low response rate (10%, inherent to survey studies) and the voluntary nature of participation, which may have caused selection bias. The fact that participation was voluntary may have led to an overrepresentation of individuals with a greater interest in environmental issues. The design of the study meant that only anaesthesiologists and nurses anaesthesiologists who were SFAR members were contacted, and the response rate was particularly low among resident anaesthesiologists, probably because of their moving between departments or hospitals every 6 months. This is unfortunate because their practices and opinions are of particular interest to better predict how motivations in this area will evolve in coming years. Finally, since only anaesthesiology teams were surveyed, this study does not provide information on the recycling behavior of other operating room staff (surgeons, healthcare assistants, operating room nurses, etc.). It provides no information either on the amounts or the types of waste that are recycled.

## Conclusion

In conclusion, our study highlights the concern of anaesthesiologists and nurses anaesthesiologists for environmental sustainability. Considerable efforts are still required to make the adoption of environmentally sustainable practices more widespread in operating rooms in France. The barriers identified here would be easy to overcome with better staff training and the creation of local environmental sustainability groups. A better integration of environmental issues in hospital policies is also required. An efficacy study of these green teams’ interventions would provide evidence for or against their use and could lead to the publication of national recommendations for the widespread creation of these groups.

## Figures and Tables

**Table 1.** Demographic Characteristics of Respondents

Characteristics	Total, n = 1092
**Role **	
Anaesthesiologist	765 (70%)
Anaesthesia nurse	264 (24%)
Anaesthesia resident	63 (6%)
**Age **	
20‐34 years	220 (20%)
35‐44 years	311 (28%)
45‐55 years	265 (24%)
>55 years	296 (27%)
**Type of hospital **	
University hospital	460 (42%)
Private hospital	267 (24%)
Regional hospital	247 (23%)
Private non-profit hospital	89 (8%)
Other	29 (3%)
**Main activity**	
Scheduled	925 (85%)
Paediatrics	91 (8%)
Emergency	76 (7%)

**Table 2.** Environmentally Sustainable Practices at Home

Personal Environmentally Sustainable Practices	Total, n = 995
Waste sorting	952 (96%)
LED lighting or energy-saving light bulbs	853 (86%)
Local consumption	797 (80%)
Lowering the thermostat	643 (65%)
Reducing packaging waste/bulk purchases	638 (64%)
Use of natural household products	633 (64%)
Reducing meat consumption	630 (63%)
Composting organic waste	498 (50%)
Cycling for short trips	419 (42%)
Energy-efficient renovation	407 (41%)
Avoiding flying	319 (32%)
Cycling to work	318 (32%)
Reduce the purchase of new clothes	229 (23%)
Buying second-hand household appliances and electronic products	196 (20%)
Supporting sustainable development associations/NGOs	167 (17%)
Use of "green" electricity or gas	145 (15%)
Carpooling	122 (12%)
Use of an electric vehicle	106 (11%)
Adopting a vegetarian diet	66 (7%)

LED, light-emitting diode; NGOs, non-governmental organizations.

**Table 3.** Forms of Sustainability Training Already Received and Desired in the Future

Form of Training	Respondents Already Trained in This Way, n = 477	Respondents Wishing to be Trained in This Way, n = 972
Local hospital training day	59 (12%)	625 (64%)
Expert recommendations	45 (9%)	517 (53%)
Online support	95 (20%)	447 (46%)
SFAR conference	192 (40%)	348 (36%)
30 minutes of scientific news (SFAR online support)	39 (8%)	333 (34%)
Articles in scientific journals	200 (42%)	216 (22%)
Conversations with colleagues	255 (53%)	143 (15%)
University course	5 (1%)	130 (13%)
Other^a^	90 (19%)	28 (3%)

SFAR, French Society of Anesthesia and Intensive Care Medecine.

^a^Anaesthesia nursing school, medical study, doctorate research.

**Table 4.** Types of Material Recycled in the Operating Room

Type of Recycled Material	n = 719
Cardboard	461 (64%)
Paper	423 (59%)
Metal	383 (53%)
Glass	290 (40%)
Batteries	247 (34%)
Plastic	193 (27%)
Copper	189 (26%)
Electronic	67 (9%)
Other	33 (5%)

**Table 5.** Current Sustainability Efforts in the Operating Room

Current Sustainability Efforts	Total, n = 1007
Turning off anaesthesia and other equipment in the evening	566 (56%)
Appropriate segregation of biohazardous and non-hazardous waste	451 (45%)
Donating unused medical equipment and supplies to medical charities	367 (36%)
Reprocessing single-use medical devices	77 (8%)
Partnering with manufacturers to promote greener packaging practices	60 (6%)
Using reusable sharps containers	24 (2%)
Unaware of any such plans	47 (5%)
Other^a^	22 (2%)

^a^Donating plastic caps to the French association "*les petits doudous,*" and eliminating the use of N_2_O, meal trays, and intravenous anaesthesia.

**Table 6.** Main Barriers to Recycling Perceived by Respondents

Main Barriers to Recycling	n = 993
Lack of information	691 (70%)
Lack of facilities	652 (66%)
Lack of support from management	602 (61%)
Lack of personnel	509 (51%)
Lack of space	474 (48%)
Staff attitudes	383 (39%)
Lack of time	282 (28%)
Cost	143 (14%)
Other	62 (6%)

## APPENDIX: SURVEY TOOL

Demographic characteristics and personal environmentally sustainable practices

1. You are:
  - Anaesthesiologist
  - Anaesthesia resident
  - Anaesthesia nurse
2. What is your age range?
  - 20-34
  - 35-44
  - 45-55
  - >55
3. What is the nearest university hospital to your hospital?
  - Amiens
  - Angers
  - Besançon
  - Bordeaux
  - Brest
  - Caen
  - Clermont-Ferrand
  - Dijon
  - Fort de France
  - Grenoble
  - La Reunion
  - Lille
  - Limoges
  - Lyon
  - Marseille
  - Montpellier
  - Nancy
  - Nantes
  - Nice
  - Nimes
  - Paris
  - Pointe à Pitre
  - Poitiers
  - Reims
  - Rennes
  - Rouen
  - Saint Etienne
  - Strasbourg
  - Toulouse
  - Tours
4. At what type of hospital do you work?
  - University hospital
  - Regional hospital
  - Private hospital
  - Private non-profit hospital
  - Other (please specify)
5. What is your primary anaesthetic activity?
  - Scheduled
  - Paediatrics
  - Emergency
6. How many years have you been in anaesthesia practice?
  - <5 years
  - 5-10 years
  - 11-20 years
  - >20 years
7. In your personal life, do you act to limit your impact environment?
  - Yes/No
8. How? (several answers are possible)
  - Supporting sustainable development associations/NGOs
  - Waste sorting
  - Composting organic waste
  - Local consumption
  - Reducing meat consumption
  - LED lighting or energy-saving light bulbs
  - Use of natural household products
  - Lowering the thermostat in your house
  - Energy-efficient renovation
  - Use of "green" electricity or gas
  - Reducing packaging waste / bulk purchases
  - Buying second-hand household appliances and electronic products
  - Reduce the purchase of new clothes
  - Cycling for short trips
  - Cycling to work
  - Avoiding flying
  - Carpooling
  - Use of an electric vehicle

Environmental sustainability initiatives in the operating room

1. To what extent do you agree with the following statement: The environmental impact of anaesthesia is an important factor that should be taken into account when conducting anaesthesia practice.
  - Strongly disagree—Disagree—Uncertain—Agree—Strongly agree
2. To what extent do you agree with the following statement: My level of knowledge on the environmental impact of anaesthesia is sufficient to guide my practice.
  - Strongly disagree—Disagree—Uncertain—Agree—Strongly agree
3. Have you already received any training/education on environmental sustainability in anaesthesia?
  - Yes/No
4. What format did this training take? (several answers are possible)
  - Local hospital training day
  - University course
  - SFAR conference
  - Expert recommendations
  - “30 minutes of scientific news” SFAR online support
  - Online Support
  - Conversations with colleagues
  - Articles in scientific journals
  - Other (please specify)
5. Is there waste sorting facilities in your operating room?
  - Yes/No/I don’t know
6. If yes, what products are recycled in your OR?
  - Paper
  - Cardboard
  - Glass
  - Plastics
  - Metal (example : laryngoscope blade)
  - Copper (example : scalpel cable)
  - Batteries
  - Electronic
  - Other (please specify)
7. Are you following your OR’s waste sorting instructions?
  - All the time/Often/Sometimes/Rarely/Never
8. Would you like to see this practice implemented or improved?
  - Yes, totally/Why not/Not at all
9. Does your operating room offer the following reusable materials?
  - Operating room caps
  - Operating room clothes
  - Metal laryngoscope blades
  - Fibroscopes
  - None
  - Other (please specify)
10. Has your anaesthesia department undertaken any other sustainability efforts? (several answers are possible)
  - Choosing anaesthetic gases based on their environmental footprint
  - Reduced fresh gas flows in anaesthesia ventilators
  - Using anaesthetic gas capture/reflection modalities
  - Reprocessing single-use medical devices
  - Using reusable sharps containers
  - Donating unused medical equipment and supplies to medical charities
  - Partnering with manufacturers to promote greener packaging practices
  - Appropriate segregation of biohazardous and nonhazardous waste
  - Turning off anaesthesia and other equipment in the evening in the OR
  - Unaware of any such plans
  - Other (please specify)
11. Personally, I take into account the environmental impact when I choose anaesthetic agents
  - All the time/Often/Sometimes/Rarely/Never
12. Personally, I take into account the impact on the environment when I choose products used to conduct anaesthetics (syringes, gloves, gowns...)
  - All the time/Often/Sometimes/Rarely/Never
13. What halogenated gas are you using? (several answers are possible)
  - Sevoflurane
  - Desflurane
  - Nitrous oxide
  - Isoflurane
14. Would you change your anaesthesia gas use habits on the sole basis of the ecological argument?
  - Yes/No
15. Most often, what fresh gas flow do you set? (unique choice)
  - 0.4-0.8 L/min
  - 0.9-1.5 L/min
  - 1.6-3 L/min
  - >3 L/min
  - Used automated end-tidal control

Barriers to and strategies for the adoption of environmentally sustainable practices

1. Which of the following do you perceive as potential barriers to recycling in the OR? (several answers are possible)
  - Staff attitudes
  - Cost
  - Inadequate information/education
  - Lack of time
  - Lack of space
  - Lack of facilities
  - Lack of support from hospital
  - Lack of staff dedicated to the implementation of these actions
  - Other (please specify)
2. Which of the following is currently the biggest barrier to recycling in the OR? (choose only one)
  - Staff attitudes
  - Cost
  - Inadequate information/education
  - Lack of time
  - Lack of space
  - Lack of facilities
  - Lack of support from hospital
  - Lack of staff dedicated to the implementation of these actions
  - Other (please specify)
3. Would you like to be more trained and aware of sustainable development practices that can be achieved in the operating room?
  - Yes, totally/Why not/Not at all
4. What type of training do you want? (several answers are possible)
  - Local hospital training day
  - University course
  - SFAR conference
  - Expert recommendations
  - “30 minutes of scientific news” SFAR online support
  - Online Support
  - Conversations with colleagues
  - Articles in scientific journals
  - Other (please specify)
5. To improve recycling in the operating room, which of the following are you willing to provide? (several answers are possible)
  - Time to educate others
  - Time to educate myself
  - Funds to education
  - Creation of environmental sustainability groups to educate staff and initiate actions
  - Other (please specify)
